# Immunohistochemical, Ultrastructural and Functional Analysis of Axonal Regeneration through Peripheral Nerve Grafts Containing Schwann Cells Expressing BDNF, CNTF or NT3

**DOI:** 10.1371/journal.pone.0069987

**Published:** 2013-08-09

**Authors:** Maria João Godinho, Lip Teh, Margaret A. Pollett, Douglas Goodman, Stuart I. Hodgetts, Iain Sweetman, Mark Walters, Joost Verhaagen, Giles W. Plant, Alan R. Harvey

**Affiliations:** 1 School of Anatomy, Physiology and Human Biology, The University of Western Australia, Crawley, Western Australia, Australia; 2 Cranio-Maxillo-Facial Unit, Princess Margaret Hospital for Children, Perth, Western Australia, Australia; 3 School of Veterinary and Biomedical Sciences, Murdoch University, Murdoch, Western Australia, Australia; 4 Netherlands Institute for Neuroscience, Amsterdam, The Netherlands; Oregon Health & Science University, United States of America

## Abstract

We used morphological, immunohistochemical and functional assessments to determine the impact of genetically-modified peripheral nerve (PN) grafts on axonal regeneration after injury. Grafts were assembled from acellular nerve sheaths repopulated *ex vivo* with Schwann cells (SCs) modified to express brain-derived neurotrophic factor (BDNF), a secretable form of ciliary neurotrophic factor (CNTF), or neurotrophin-3 (NT3). Grafts were used to repair unilateral 1 cm defects in rat peroneal nerves and 10 weeks later outcomes were compared to normal nerves and various controls: autografts, acellular grafts and grafts with unmodified SCs. The number of regenerated βIII-Tubulin positive axons was similar in all grafts with the exception of CNTF, which contained the fewest immunostained axons. There were significantly lower fiber counts in acellular, untransduced SC and NT3 groups using a PanNF antibody, suggesting a paucity of large caliber axons. In addition, NT3 grafts contained the greatest number of sensory fibres, identified with either IB_4_ or CGRP markers. Examination of semi- and ultra-thin sections revealed heterogeneous graft morphologies, particularly in BDNF and NT3 grafts in which the fascicular organization was pronounced. Unmyelinated axons were loosely organized in numerous Remak bundles in NT3 grafts, while the BDNF graft group displayed the lowest ratio of umyelinated to myelinated axons. Gait analysis revealed that stance width was increased in rats with CNTF and NT3 grafts, and step length involving the injured left hindlimb was significantly greater in NT3 grafted rats, suggesting enhanced sensory sensitivity in these animals. In summary, the selective expression of BDNF, CNTF or NT3 by genetically modified SCs had differential effects on PN graft morphology, the number and type of regenerating axons, myelination, and locomotor function.

## Introduction

Peripheral nerve (PN) injuries are often microsurgically repaired by coaptation of transected nerve stumps. However if the nerve defect is too large, due to nerve stump retraction or following pruning to remove necrotic tissue, a bridging graft is needed to restore continuity. Autologous nerve grafts are the preferred option, commonly harvested from sensory sural nerves [1], [2], yet functional recovery can be suboptimal, perhaps due to neuronal loss, deterioration of distal nerve stump, or failure to recruit Schwann cells (SCs) of the appropriate phenotype [3]–[6]. Moreover, harvesting autografts may result in functional impairment and neuroma formation at the donor site. Use of allograft or xenograft material requires immunosuppression, and graft rejection results in axonal loss [7]–[9]. Alternative substrates include muscles, tendons and veins, although none have yet matched the performance of autografts [10], [11]. Bridges using synthetic materials have the advantage of ease of fabrication and availability, although they may not be optimal for repairing large nerve defects and may induce inflammatory reactions [12].

An approach that may potentiate regeneration and minimize adverse effects is to develop chimeric grafts composed of optimized support structures, cell types and molecules [2], [13], [14]. For example, because cells in PN tissues are the primary immunogenic component [9], [15], [16], and the essential PN structure and organization is maintained after freeze-thawing, it is possible to repopulate allogeneic acellular PN sheaths *ex vivo* with cultured, congeneic SCs that support axonal regeneration *in vivo* [17], [18]. Furthermore, after PN injury, neuronal survival and axonal regrowth is enhanced by administration of neurotrophic factors delivered systemically [10] or locally using osmotic pumps [19]–[21]. Neurotrophic factors have also been delivered by direct injection into the PN of viral vectors encoding these factors [22]–[24] or *in vivo* injection of genetically modified SCs [25]. The former technique results in transduction of diverse cell types, including not only SCs but also fibroblasts and endothelial cells [26], [27].

Here we used an alternative method for local neurotrophic delivery, shown previously to promote successfully the regrowth of injured axons in the adult rat visual system [28], [29]. Our aim was to compare the effects of different neurotrophic factors on various aspects of regeneration through PN bridging grafts. Purified adult SCs were transduced *ex vivo* using lentiviral (LV) vectors to express either brain-derived neurotrophic factor (BDNF), a secretable form of ciliary neurotrophic factor (CNTF), or neurotrophin-3 (NT3). Genetically modified SCs were then injected into cell-free PN sheaths and 24 hr later the reconstituted grafts inserted into a unilateral 1 cm gap in adult rat peroneal nerves. This gap size allows for direct comparison of the effects of each neurotrophic factor on axonal regeneration and myelination, while minimising the impact related to the length of the nerve defect itself [1]. For comparison, uninjured peroneal nerves, autografts, acellular grafts, and grafts containing unmodified SCs cultures were also examined. In behavioral studies we compared walking patterns prior to, and 1 and 8 weeks after surgery using the Ratwalk® gait analysis system, a software written and developed independently but which analyzes parameters similar to those previously described in the Catwalk system [30]. Graft morphologies were compared 10 weeks post-transplantation, and the number and type of regenerating axons were analyzed using immunohistochemistry. The number, distribution and extent of myelination of regenerate axons were also quantified in semi- and ultra-thin sections.

## Materials and Methods

### SC cultures

Sciatic nerves from young adult male Fischer 344 rats were used as the source for SCs, which were isolated and purified using established protocols [31], [32]. For each culture, 5 animals were overdosed (sodium pentobarbitone, Lethabarb), their sciatic nerves collected and placed in Liebovitz's L-15 medium (Invitrogen). Sciatic nerves were chosen for their high SC yield and because they are a mixed nerve. This is important because there is evidence that SCs “express distinct sensory and motor phenotypes that are associated with the support of regeneration in a phenotype-specific manner” [4]. Nerves were stripped of epineurium, sectioned into 1–2 mm pieces and incubated at 37°C with 5% CO_2_ in culture dishes with D-10 media (Dulbecco's Modified Eagle's Medium (DMEM) (Sigma) containing 10% foetal bovine serum (FBS) (Sigma), 1% L-glutamine (Invitrogen) and 1% penicillin/streptomycin (Invitrogen). Fibroblasts migrated out of the PN explants, the latter transferred weekly and plated cells discarded. After 3–4 weeks, explants were dissociated overnight with 1.25 U/mL dispase (Boehringer Mannheim Biochemicals) and 0.05% collagenase (Sigma) in DMEM with 15% FBS. SCs cultures were expanded on poly-L-lysine (Sigma) coated dishes in D-10 media containing 20 µg/mL bovine pituitary extract (GibcoBRL) and 2 µM forskolin (Sigma).

### Lentiviral vectors and SCs transduction

Genetic modification of SCs using LV was approved by the Office of Gene Technology Regulator, Australia. The LV constructs used for expression of neurotrophic factors were previously characterized, and expression and release of neurotrophic factors in SCs and peripheral tissues confirmed, both *in vitro* and *in vivo* [22], [23], [26], [28], [29]. In brief, cDNA encoding either a rat CNTF fragment (which contained the signal sequence required for the release of human growth hormone), BDNF or NT3, were cloned into the LV transfer vector backbone pRRLsin-PPThCMV-MCS-wpre and LV stocks produced by co-transfection of the vector, packaging and envelope plasmids into 293T cells. Titers were between 10^8^ and 10^9^ transducing units/ml. About 10^6^ SCs were plated for 24 hr prior to transduction with LV-BDNF, LV-CNTF or LV-NT3 at a multiplicity of infection of 50. After 24 hr, the D10 medium was refreshed and cells incubated for 48 hr to allow for maximum transgene expression. In a preliminary sciatic nerve graft experiment, LV encoding green fluorescent protein (GFP) was used to verify SC viability and sustained transgene expression after injection of transduced cells into grafts and transplantation into a PN injury site.

### Acellular nerve sheaths

To create acellular nerve sheaths, cells were eliminated by 5 consecutive cycles of 5 min immersion in liquid nitrogen and 5 min thawing at room temperature and storage at −80°C. The freeze-thaw cycles killed the cells but maintained basal lamina integrity, providing flexible nerve sheaths that can be effectively repopulated with cultured cells [17], [18]. Acellular sheaths were prepared from either sciatic or peroneal nerves of adult male Wistar rats and inserted into the corresponding nerve in Fischer 344 host rats.

### Cellular reconstitution of genetically modified nerve sheaths

Three days after transduction, cultured SCs were rinsed twice with Ca^2+^ and Mg^2+^ free Hanks balanced salt solution (Sigma) and detached from plates by incubation for 5 min at 37°C with 0.02% EDTA (Invitrogen) and 0.05% trypsin (CSL), then inactivated with D10. Cells were collected by centrifugation at 1000 rpm and resuspended in D10 to a final concentration of 5×10^4^/µl [18]. Acellular nerve sheaths were placed in D10, trimmed to 1 cm length and 1 µl of the SC suspension slowly injected via a glass micropipette using a Hamilton syringe into both ends of each nerve sheath, giving an approximate concentration of 10^5^ SCs/sheath. This number of cells has previously been shown to result in complete colonization of the nerve sheaths [18], [28], [29]. To allow further SC infiltration, a small amount of cell suspension was placed around each nerve and further incubated for 24 hr.

### Ethics Statement

Surgical procedures followed NHMRC guidelines and the study was approved by the University of Western Australia Animal Ethics Committee (approval RA3/100/471). Rats were obtained from the Animal Resource Centre, WA, and housed under standard conditions with a 12 hr light/dark cycle and *ad libidum* access to food and water. All surgical procedures were performed under anaesthesia and animals received antibiotic treatment (Benacillin, 200 µl/100 g) to reduce the risk of infections and an analgesic (Temgesic, 20 µg/kg) to minimize post-operative discomfort.

### Host animals

Adult (8–10 weeks old) male Fischer 344 rats received a unilateral 1 cm peripheral nerve cut, either on the sciatic nerve in the preliminary study, in which we assessed SC survival and the continued expression of transgenes at 8 weeks following transplantation, or on the peroneal nerve in the main study. The 1 cm gap was then repaired using different types of chimeric nerve grafts. Experimental groups received acellular nerve sheaths repopulated with SCs genetically modified with LV to over express BDNF, CNTF or NT3, while control groups received acellular nerve sheaths with unmodified SCs, acellular nerve sheaths without any cells, or autografts. The normal control group included uninjured rats that were processed to obtain intact peroneal nerve material. In a preliminary experiment, 3 rats received acellular sciatic nerve sheaths repopulated with SCs transduced with LV-GFP to verify long-term transduction. Given that SCs rapidly migrate in and out of PN to grafts [33], labelling of transplanted SCs with GFP made it possible to distinguish donor from host SCs, allowing an assessment of their viability and distribution after surgery.

### Surgical injury model and tissue collection

Each host rat was anesthetized with an intra-peritoneal injection (1 mL/kg body weight) of a mixture of equal volume of ketamine (100 mg/mL) and xylazine (20 mg/mL). Either the sciatic (preliminary study – to confirm SC viability and long-term transgene expression after transplantation) or the peroneal nerve (main study – to investigate the effects of overexpressing different neurotrophic factors on axonal regeneration), in the left hind limb was exposed and a 1 cm segment was removed. The nerve gap was repaired with different types of grafts, all attached to host nerve stumps using 10/0 nylon suture (Ethicon), and the injury closed with 6/0 suture (Ethicon). Animals received Benacillin (200 µl/100 g of body weight) intra-muscularly and Temgesic (20 µg/kg body weight) subcutaneously as post-operative care.

**Figure 1:**
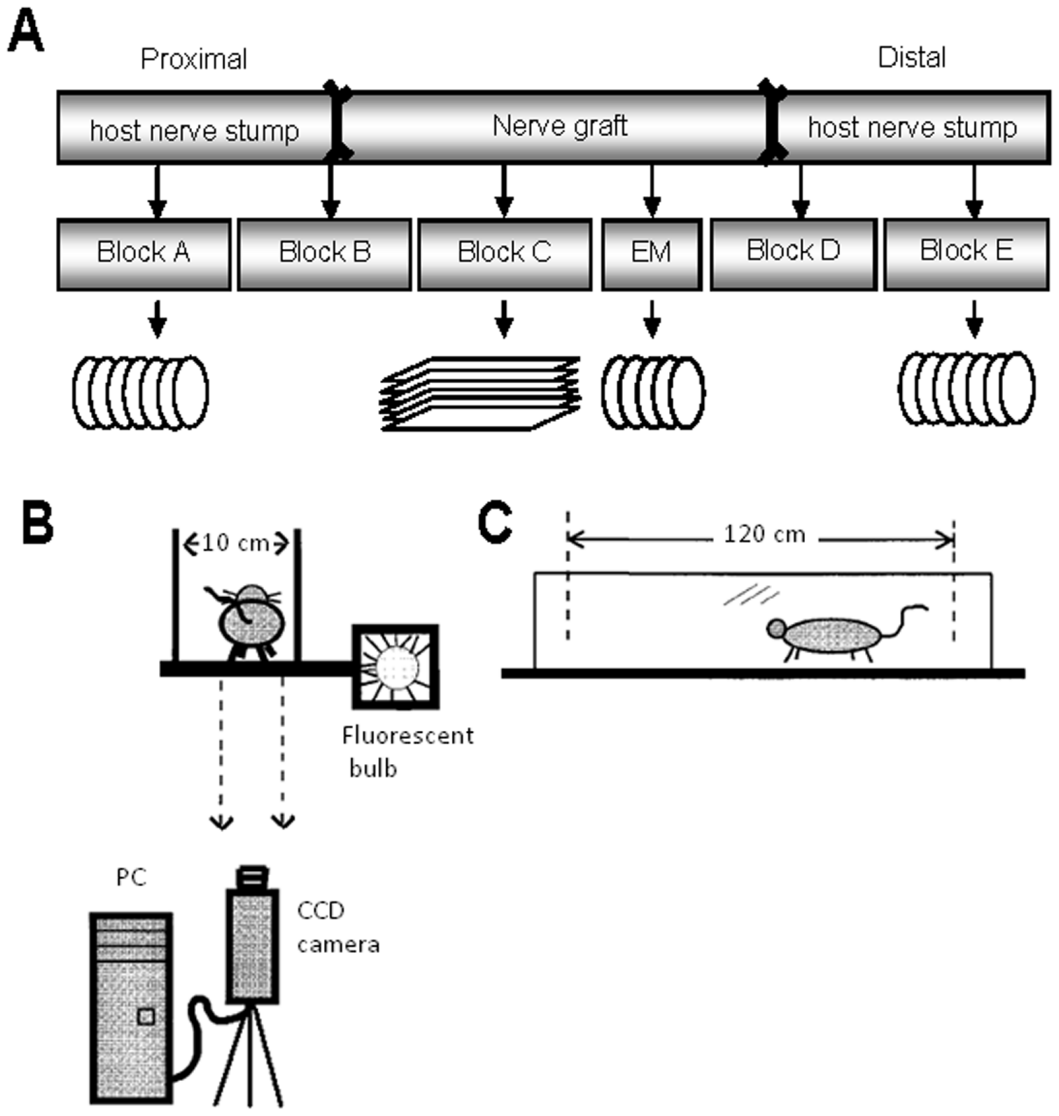
Tissue sampling protocol and Ratwalk® schematic. (A) Each grafted nerve was divided into blocks: proximal host nerve stump (Block A), proximal suture (Block B), graft itself (Block C), distal suture (Block D), and distal host nerve stump (Block E). Either cross sections (circles) from Blocks A and E or longitudinal sections (rectangles) from Block C were collected for immunohistochemistry. Additionally, a block was taken from the distal end of 3 grafts from each group to collect semi- and ultra-thin cross-sections for electron microscopy (EM). (B, C) Functional recovery was assessed using the Ratwalk® [side view of the setup (B)], consisting of a glass walking platform [front view (C)] attached to a box containing a fluorescence bulb from which the light escapes only through a narrow slit into the glass. Light is scattered from the glass at each point of contact with the animals' paws. Walks were recorded and analyzed using the Ratwalk® software (images B and C modified from 30).

Ten weeks after surgery (8 weeks in preliminary study) grafted rats received a lethal dose of sodium pentobarbitone (Lethabarb, 325 mg/ml, intra-peritoneal). Fresh grafted nerves were collected, gently straightened, attached to a wooden spatula and fixed in 4% paraformaldehyde for 3 hr at 4°C, after which a 1 mm block was taken from the distal end of 3 grafts from each group. These samples were placed in 2% glutaraldehyde and processed for electron microscopy, while remaining tissue was cryoprotected in 30% sucrose solution for 24 hr at 4°C. To analyze distinct nerve regions, samples were divided into 5 blocks (Fig. 1A). Each block was embedded in tissue freezing medium (Leica), snap frozen in isopentane (2-methylbutane) and stored at −80°C. Frozen blocks from the host proximal (Block A) and distal (Block E) nerve stumps were sectioned at 16 µm thickness using a Leica CM3050 cryostat into series of 7 slides, each with 8 cross-sections. A series of 12 slides was prepared by cutting grafts (Block C) into 10 µm thick longitudinal sections, resulting in 7–12 sections per slide, depending on the graft. Sections were collected onto gelatine coated slides, air-dried and stored at −20°C.

### Immunohistochemical staining of cryosections of nerves

Slides were placed in a humidified dark chamber at room temperature with gentle agitation. Sections were rinsed with PBS (3×5 min), blocked for 1 hr in PBS with 10% normal horse serum and 0.2% Triton X-100, and incubated overnight in primary antibodies. This incubation was done at 4°C for antibodies to axonal neurofilaments (PanNF) (Invitrogen #18-0171Z, 1∶500), neuronal class III β-Tubulin (Covance #MMS-435P, 1∶400), S100 (DAKO #Z0311, 1∶500), myelin basic protein (MBP) (Abcam #120.24040, 1∶100), macrophages (ED1, Millipore MAB1435, 1∶500), or laminin (Sigma #L9393, 1∶400). Immunostaining was done at room temperature for antibodies to calcitonin gene-related peptide (CGRP) (AbDSerotec #1720-9007, 1∶1000) to label axons of primary peptidergic sensory neurons. Non-peptidergic sensory axons were identified by histochemical reaction with fluorescently labelled isolectin B_4_ (IB_4_) (Vector Laboratories, 1∶100) [34]. Each immunostaining run included a negative control without primary antibody. After 3×5 min washes with PBS, appropriate secondary antibody dilutions were added: goat anti-mouse Cy3 (Jackson Immuno/research Labs #115-166-006, 1∶500); goat anti-rabbit Cy3 (Jackson Immuno/research Labs #111-166-006, 1∶300); donkey anti-goat Cy3 (Jackson Immuno/research Labs #705-166-147, 1∶1000); goat anti-rabbit FITC (Sigma F6005, 1∶100); and rabbit anti-goat FITC (sigma F7367, 1∶100). Sections were washed with PBS, mounted with citifluor or fluorescence mounting medium (DAKO), and cover-slipped. Stained sections were kept at 4°C.

### Quantification of axonal numbers

Counts of βIII-Tubulin^+^ axons were made from cross-sections of proximal and distal host nerve stumps. Sections were photographed using a 10× objective and a QuantiFIRE camera operated by PictureFrame™ software (Optronics). The outline of each section was manually traced using Image-Pro Plus software (MediaCybernetics) and axonal number quantified using a filter algorithm plugged to the software [23]. The number of regenerating axons was also quantified in longitudinal graft sections immunohistochemically stained for βIII-Tubulin or PanNF. In sections stained with βIII-Tubulin, 3 photographs were taken and on each of them a line was placed to mark 1.2, 2.4 and 3.6 mm from the proximal end of the section. The number of axons crossing these lines was counted and the section width was measured. Sections stained with PanNF, IB_4_ and CGRP were photographed only at the middle location of βIII-Tubulin analysis (i.e. 2.4 mm from the proximal edge of the section), and axon numbers and section width measured and quantified (Image-Pro Express software, MediaCybernetics). Given that IB_4_ labels not only small, nonpeptidergic, unmyelinated, sensory nociceptive neurons and their axons [34] but also endothelial cells [35]–[37], the marker PanNF was used to check that IB_4_ labelling was associated with clearly defined axonal profiles.

### Statistical analysis of axonal counts

Statistical analyses were carried out using PASW software following Pallant guidelines [38]. Descriptive statistics were done and mean values (M) and standard error of the mean (SEM) were plotted. The size of each sample (n) is indicated, and corresponds to the number of animals within each group. Parametric tests were used preferentially and logarithmic or square root transformations were applied to data in order to correct for non-normality. Parametric tests included linear mixed models, with a fixed effect of experimental group and block and a random effect of animal within group and also including analysis of interactions between variables [39], using a Sidak adjustment to account for multiple comparisons, and one-way analysis of variance, followed by least significant difference (LSD) test when significant differences were found between groups. Non-parametric Kruskal-Wallis tests followed by Dunn's post-hoc analysis were performed with all pairwise comparisons when significant differences were found between groups, and median (Md) values calculated. For all tests, significance levels were set at p<0.05, although Bonferroni corrections were applied whenever multiple tests were carried out on the same data. Note that the specific statistical analyses selected for each parameter are presented in legends to relevant Figures and Tables, and are explained in detail in Statistical Information S1.

### Quantification of axons in semi-thin sections

From the distal end of Block C of 3 grafts in each group a 1 mm block was collected and fixed in 2% glutaraldehyde. Further processing, including bright field photography of semi-thin plastic sections, was done by an independent technician and all subsequent counts were done blind. Using Image-Pro Express 5.1 software, images were digitally zoomed and myelinated profiles were counted within 4 areas of 4004 µm^2^ in each section. The number of myelinated axons/mm^2^ was calculated and assessed using one-way analysis of variance. The number of samples and the power of each statistical test are indicated in the results. In addition, in a semi-thin section from three nerves in each of the SCs, BDNF, CNTF and NT3 graft groups, the diameter of fascicles was measured and compared using ANOVA and Bonferroni post-hoc test.

### Analysis and quantification in ultrathin sections

Electron micrographs were collected in 5 different locations on each ultra-thin section. Locations were selected in a systematic but random manner and files identified by a code so that the origin of each sample was not known. From each location, one set of micrographs was used to quantify all myelinated and unmyelinated axons within an area of 325 µm^2^, as well as the number of unmyelinated axons in Remak bundles. Only those axons that were entirely within the image were quantified. Ratios between unmyelinated and myelinated axons were calculated. Another set of micrographs was used to measure the area and diameter of axons and fibers, the latter including the myelin sheath. The difference between the total area of each fiber and the area of the axon provided the myelin area. The diameter of each myelinated axon was divided by the diameter of the entire fiber to calculate the G-ratio. All data were first analyzed to determine M, SEM, variance and range of distribution with minimum and maximum values, and percentile distributions including Md. Data were then compared using Kruskal-Wallis tests followed by a Dunn's post-hoc tests with all pairwise comparisons (significance level, p<0.05). Frequency plots were used to display all data, except G-ratios, which were plotted against axonal diameter.

### Analysis of locomotor function

Functional recovery was assessed using the Ratwalk® methodology, a computerized gait analysis system similar in principle to other systems [30], [40], that quantifies several locomotor parameters (Fig. 1 B, C). Initially animals were allowed to familiarize with the setup during 3 training sessions, after which videos of animals walking along a glass platform were recorded 2 days before surgery, to establish baseline values, and then 1 and 8 weeks after surgery. Analysis of the recordings was done blinded to the group to which the animal belonged. Each recording was loaded into the Ratwalk® software, cropped to eliminate redundant frames, usually at the beginning and end of the recording where no paws could be seen, and in each frame of the cropped video all paw prints were manually identified and labelled. Data was saved as a composite file and the software quantified several gait parameters. Details on the statistical analyses are explained in Statistical Information S1.

Given that the Ratwalk® was originally developed to evaluate gait recovery following spinal cord injuries we used it in a preliminary study (data not shown) to assess which of the output parameters were more suitable for the evaluation of locomotor function after PN injuries. Two parameters are presented here: (i) stance width, which considers the mean distance between the right and left forelimb (rf-lf) and between the right and left hindlimb (rh-lh); (ii) step length, which is the measurement between ipsilateral limbs, that is, the mean distance between placements of the rf in relation to the rh and the mean distance between placements of the lf in relation to the lh. Analysis included recordings from 4 animals from groups with neurotrophic factor-delivering grafts (BDNF, CNTF and NT3) and the SC group as control. Distances between limbs in all graphs are expressed in pixels. Data were averaged for each group and analyzed using a multivariate analysis of variance, including Box's M Test of Equality of Covariance Matrices (using p = 0.001) and Levene's Test of Equality of Error Variances (using p = 0.05) to check that experimental data satisfied required assumptions for the analysis, with LSD post-hoc tests performed to specifically identify significant differences.

## Results

### Transgene expression and survival of transplanted SCs

**Figure 2:**
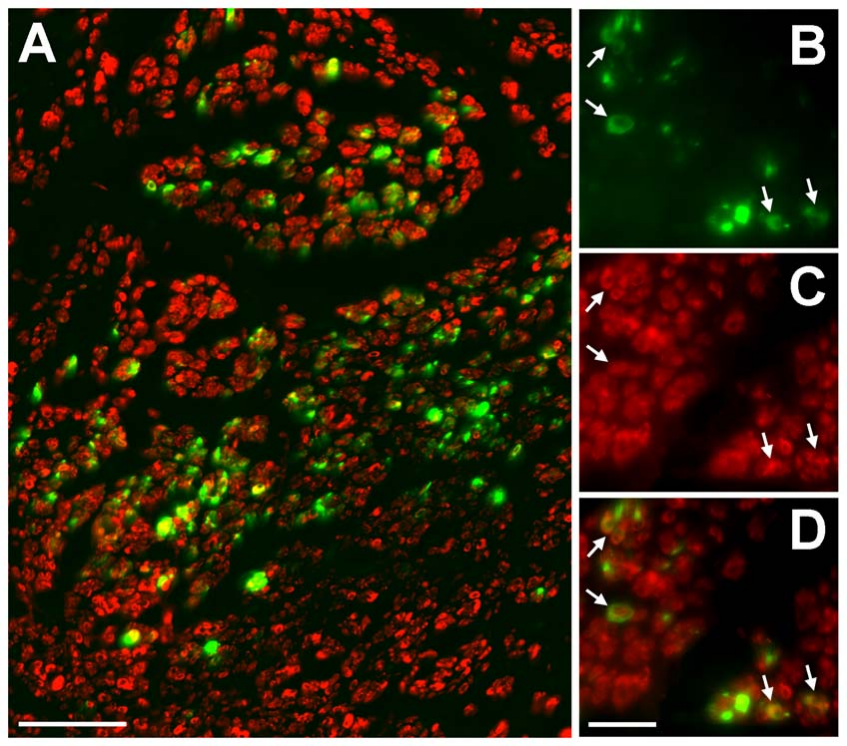
Survival of Schwann cells in reconstituted peripheral nerve grafts. (A) Low power view showing GFP and S100 positive cellular profiles in a section from the middle of a SC-GFP graft. Higher power views demonstrate: (B) continued GFP transgene expression in grafted SCs; (C) cells positively immunostained with the SC marker S100; (D) arrowed GFP and S100 double labelled cells in a combined image. Scale bars: A = 50 µm; B–D = 20 µm.

Using an LV-GFP construct we first showed sustained transgene expression in many SCs for up to 8 weeks after transplantation of reconstituted PN grafts into injured sciatic nerve (Fig. 2A). Double-labelling of sections with S100 confirmed the identity of grafted GFP cells (white arrows in Fig. 2B–D). GFP negative SCs were also seen, many presumably host SCs that had migrated into the grafts [33]. Thus as described previously [18], [28], LV-modified SC remained viable in the grafts for many weeks and can be used as vehicles to provide sustained delivery of transgene-derived factors within transplanted chimeric PN [28], [29].

### Axonal quantification in cross-sections of proximal and distal host nerve stumps

**Figure 3:**
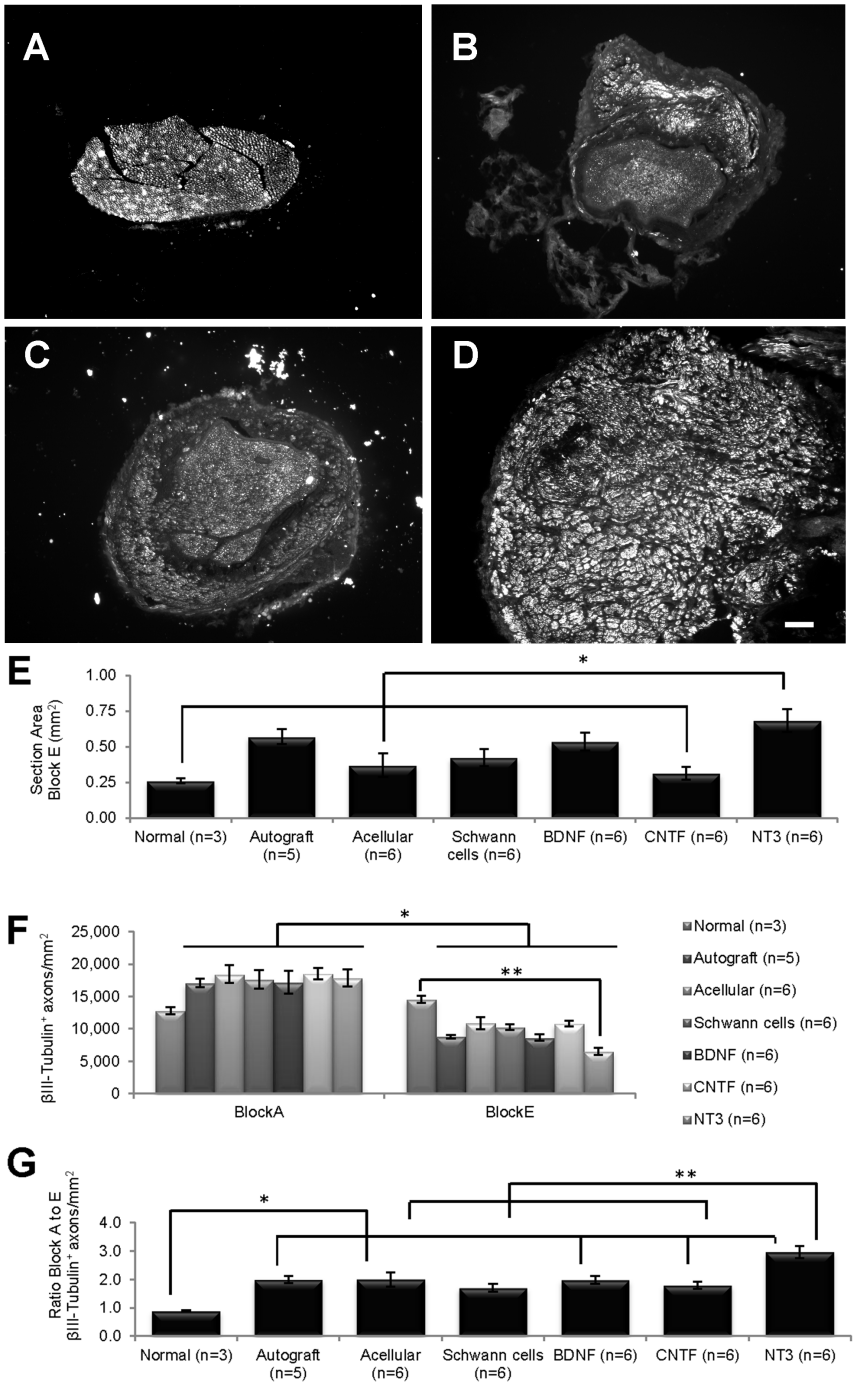
βIII-Tubulin^+^ immunostaining in nerve cross-sections. (A–D) Cross-sections of normal nerve (A) and of distal host nerve stumps of acellular (B), CNTF (C) and NT3 (D) grafts. (E) Cross-sectional areas of NT3 grafts were significantly larger (*) than areas of normal nerves and of acellular and CNTF grafts. (F) The density of βIII-Tubulin^+^ axons/mm^2^ in all grafted nerves was significantly greater (*) in proximal (Block A) than in distal (Block E) stumps, and greater (**) in the normal versus NT3 group in Block E. (G) The ratio of βIII-Tubulin^+^ axons/mm^2^ between nerve stumps was significantly lower in normal nerves than in grafts (*), except those with unmodified SCs. The ratio in the NT3 group was significantly greater (**) than in acellular, SCs and CNTF groups. Values represent M ± SEM; p<0.0005 in E and G and p<0.05 in F. Further details on statistical analysis provided as Statistical Information S1. Scale bar for A–D: 100 µm.

In the main study, primary SC cultures were transduced with LV-BDNF, LV-CNTF or LV-NT3 and seeded into acellular nerve sheaths to bridge a 1 cm peroneal nerve defect. This size gap permits an analysis of the relative impact of each neurotrophic factor on axonal regeneration and myelination, and reduces any confounding effect related to the length of the nerve defect itself [1]. Grafted nerves were collected after 10 weeks, and along with normal nerves and other control groups, divided into 5 blocks for immunohistochemical analysis (Fig. 1A). Axons were highly disorganized close to host-graft suture areas (Blocks B and D) and impossible to count, hence we focused on the central portion of each graft (Block C), and the proximal (Block A) and distal (Block E) host nerve stumps, because they reflect the number of neurons surviving injury and the number of axons potentially reinnervating target tissues, respectively. Cross-sectional areas from Block A (normal nerves or proximal host nerve stump) did not differ between groups. However there were significant differences between section areas in Block E (normal nerves or distal host nerve stump) (Fig. 3A–E), the nerve area of NT3 grafts being significantly larger than the areas of normal nerves, and acellular and CNTF grafts.

βIII-Tubulin^+^ axons in cross-sections of proximal (Block A) and distal (Block E) host nerve stumps and normal PN were counted and the density of axons/mm^2^ estimated. There was a significant interaction between groups and blocks (p<0.0005), meaning that one variable has an effect on the other; specifically, there was a significant effect associated with blocks (p<0.005), but no effect associated with experimental groups. In all grafted nerves there was a decrease in the average density of βIII-Tubulin^+^ fibers between Blocks A and E (Fig. 3F). Moreover, while there were no significant differences between groups in Block A, in Block E pairwise comparisons revealed a considerably lower density of axons in NT3 compared to normal PN.

For each group, the density of βIII-Tubulin^+^ axons/mm^2^ in Block A (proximal stump) was divided by the number in Block E (distal stump) to obtain a ratio between the two locations. Values greater than one reflect a higher density of axons proximal versus distal. As expected, the ratio in normal nerves was close to one. However, there were disparities in graft groups, and the average normal nerve ratio differed significantly from the ratios in all graft groups apart from the unmodified SC group. Moreover, the Block A/E ratio in NT3 grafts was significantly greater than in acellular, SC and CNTF groups, consistent with the increased area of the distal host peroneal stumps in NT3 grafted animals (Fig. 3G).

### Axonal quantification with βIII-Tubulin in longitudinal sections of grafts

**Figure 4:**
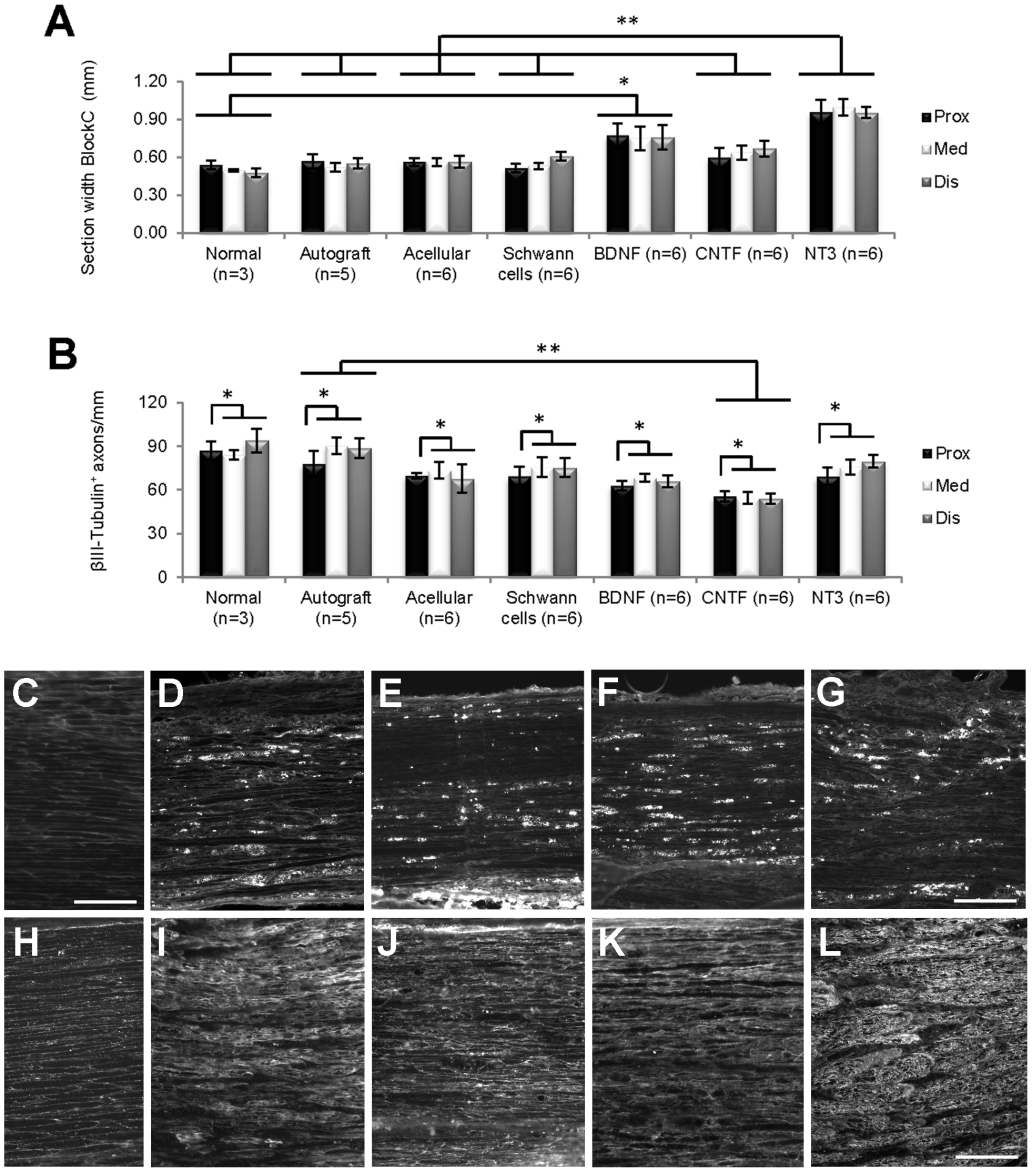
Analysis of longitudinal graft sections. (A) Within the grafts themselves (Block C), longitudinal sections of BDNF grafts were significantly wider (*) than normal nerves, and sections of NT3 grafts were significantly wider (**) than those of normal nerves, autograft, acellular, SCs and CNTF grafts. (B) Overall, the number of βIII-Tubulin^+^ axons/mm in longitudinal sections differed between the proximal and other counting distances (*), with sections of autografts containing significantly more axons (**) compared to CNTF grafts. Values represent M ± SEM; p<0.025 in A and p<0.05 in B. Further details on statistical analysis provided as Statistical Information S1. (C–G) ED1 immunostaining; C normal PN; D–G, acellular, BDNF, CNTF and NT3 grafts respectively. (H–L) laminin immunostaining; F, normal PN; G–L, acellular, BDNF, CNTF and NT3 grafts respectively. Scale for D–G = 200 µm, for C, H–L = 100 µm.

**Figure 5:**
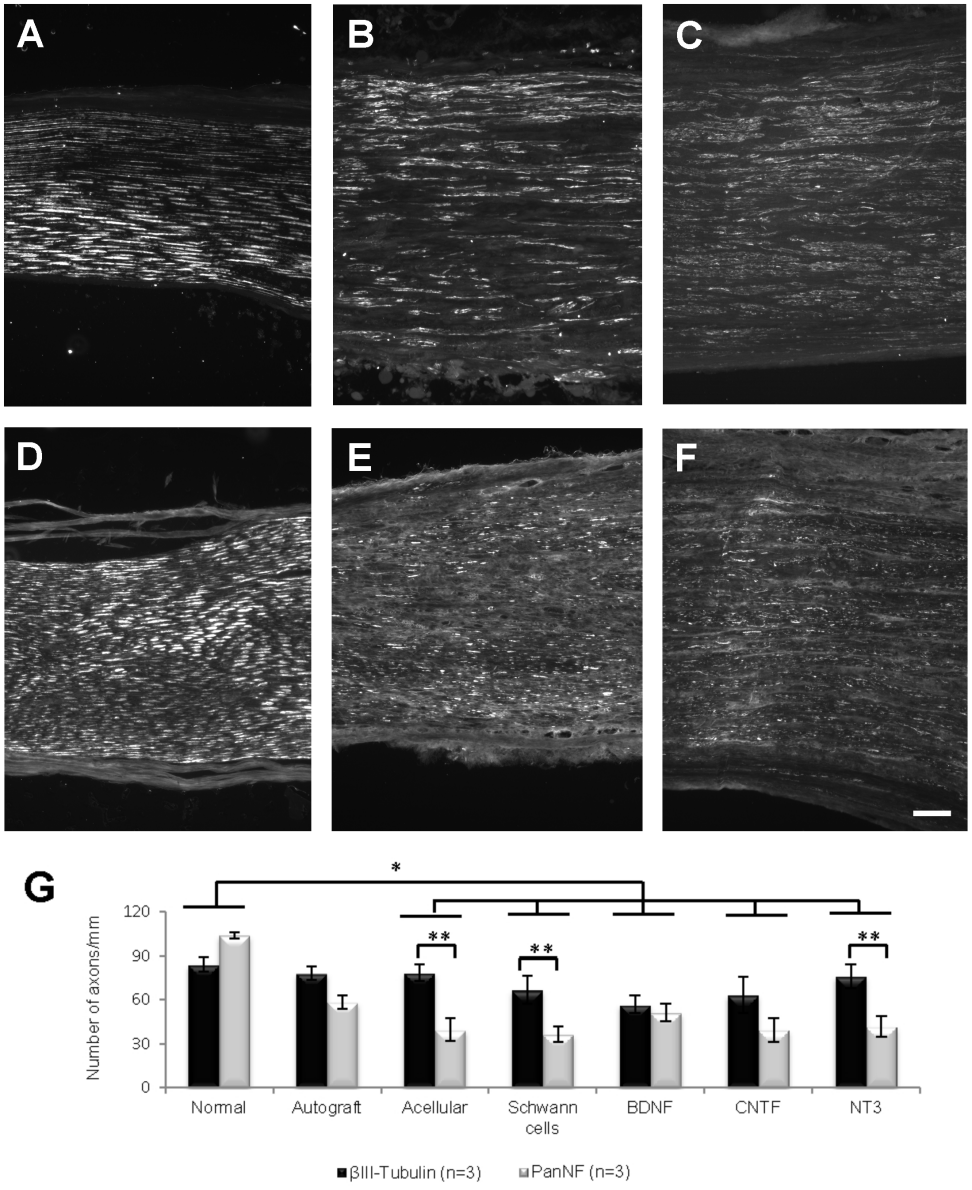
Representative examples of longitudinal sections of normal nerve (A and D), SCs (B and E) and NT3 (C and F) grafts immunostained with βIII-Tubulin (A–C) or PanNF (D–F). Images are series of sections from the same nerve or graft. (G) The difference between βIII-Tubulin^+^ and PanNF^+^ axons/mm in the normal group was significantly different from that observed in all groups except the autograft group (*). The numbers of axons/mm identified by each axonal marker were significantly different (**) in the acellular, SCs and NT3 groups. Values represent M ± SEM and p<0.05. Further details on statistical analysis provided as Statistical Information S1. Scale bar for A–F: 100 µm.

Longitudinal sections from normal PN and Block C (exclusively graft tissue, Fig. 1) were immunostained with βIII-Tubulin and the graft width measured at 3 separate locations in each section, proximal to distal from the CNS (Fig. 4A). Within each group there was no difference in section width along the length of the grafts, but section width differed significantly *between* groups (Kruskall-Wallis, p<0.0005). BDNF grafts were significantly wider than normal nerves, and sections of NT3 grafts wider than sections taken from normal nerves, autograft, acellular, SCs and CNTF grafts. In each section, the number of immunostained βIII-Tubulin^+^ axons/mm (see Fig. 5B,C) was counted at each location (Fig. 4B). There was no interaction between group and distance, indicating that these variables were not affecting each other, but there was an effect of both group (p = 0.020) and distance (p = 0.018). Namely, there were significantly more βIII-Tubulin^+^ axons/mm in the autograft compared to CNTF group, and there were significant differences in the number of βIII-Tubulin^+^ axons/mm in proximal versus both middle and distal locations, usually with fewer axons counted proximally.

A qualitative inspection of sections immunostained for the macrophage/monocyte marker ED1 revealed very few cells in normal PN (Fig. 4C), but increased aggregations of ED1 positive profiles in grafts (Fig. 4D–G), particularly in acellular (Fig. 4D) and CNTF (Fig. 4F) grafts. Again, compared to normal PN (Fig. 4H), there was increased expression of laminin in all grafts (Fig. 4I–L), especially noticeable in acellular grafts (Fig. 4J) and grafts initially seeded with NT3 expressing SCs (Fig. 4L). The fascicular nature of laminin immunostaining was a characteristic feature of NT3 grafts.

### Axonal regeneration in grafts assessed using PanNF, IB_4_ and CGRP antibodies

#### PanNF

Graft sections adjacent to those processed for βIII-Tubulin were immunostained using a PanNF antibody. In normal PN, as expected, staining patterns with either antibody appeared similar (Fig. 5A and 5D). However this was not the case in grafts. Staining in βIII-Tubulin immunoreacted sections was comparatively more homogenous, and long thick axons were intensely stained (Fig. 5B and 5C), whereas in PanNF immunoreacted sections the axons were more diffuse and appeared to be more randomly organized (Fig. 5E and 5F). PanNF^+^ profiles/mm were counted at the same location used for βIII-Tubulin quantification and compared with the number of βIII-Tubulin^+^ axons/mm using a linear mixed model including the interaction between group and axonal marker as a fixed effect. Both group and antibody had significant effects (respectively, p = 0.015 and p = 0.001). Pairwise comparisons revealed that the difference in axon counts between βIII-Tubulin and PanNF antibodies in normal PN differed significantly from the difference between these antibodies in all other groups, except autografts. Compared to PanNF there were significantly more βIII-Tubulin^+^ axons/mm in acellular, SC and NT3 grafts (Fig. 5G).

#### IB4

**Figure 6:**
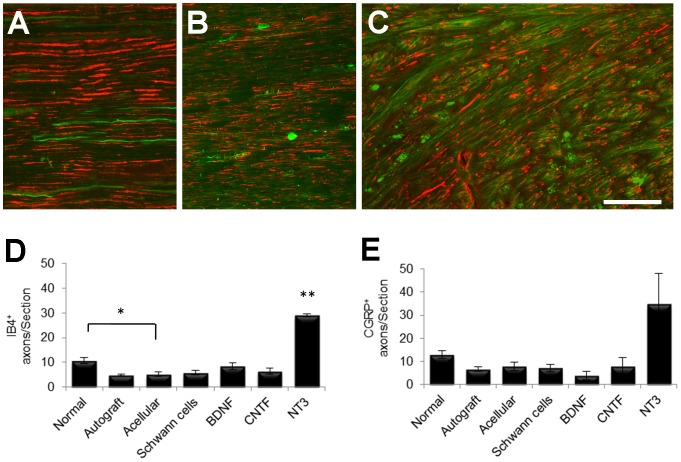
Examples of longitudinal sections of normal (A), autograft (B) and NT3 graft (C) stained with PanNF (red) and IB_4_ (green). (D) Quantification revealed significantly greater numbers of IB_4_^+^ axons in normal nerves compared to acellular grafts (*) and in NT3 grafts compared to all other experimental groups (**). (E) The number of CGRP^+^ axons was not significantly different between experimental groups. Values represent M ± SEM of n = 3; p<0.05. Further details on statistical analysis provided as Statistical Information S1. Scale bar for A–C: 100 µm.

To further characterize the type of regenerate axons within grafts, longitudinal sections were immunostained with IB_4_, a marker for small, nonpeptidergic, unmyelinated, sensory nociceptive neurons (Fig. 6A–C). Because this marker is also reported to label endothelial cells [35]–[37], only longitudinally oriented (and clearly not cellular) IB_4_^+^ profiles that were seen in close association with PanNF^+^ axons were counted. Counts were made at similar locations to those selected for the previous βIII-Tubulin and PanNF counts, revealing significant differences between groups (p = 0.001). The lowest number of IB_4_^+^ profiles was seen in autografts (Fig. 6B) and in acellular grafts (not shown). Subsequent post-hoc comparisons using LSD revealed significantly more IB_4_^+^ axons in normal PN than in acellular grafts, and more axons in NT3 grafts than in any other group (Fig. 6C,D).

#### CGRP

Another series of longitudinal graft sections was immunostained with a CGRP antibody in order to identify axons of peptidergic, unmyelinated, nociceptive sensory neurons [23], [41]. CGRP^+^ axons in normal and grafted nerves were counted at the same location (middle of graft) as other axonal counts. There was considerable variance and no significant difference between groups was found, despite a trend to greater numbers of CGRP^+^ axons in sections of NT3 compared to other groups (Fig. 6E).

### Fascicular organization of normal and grafted nerves

**Figure 7:**
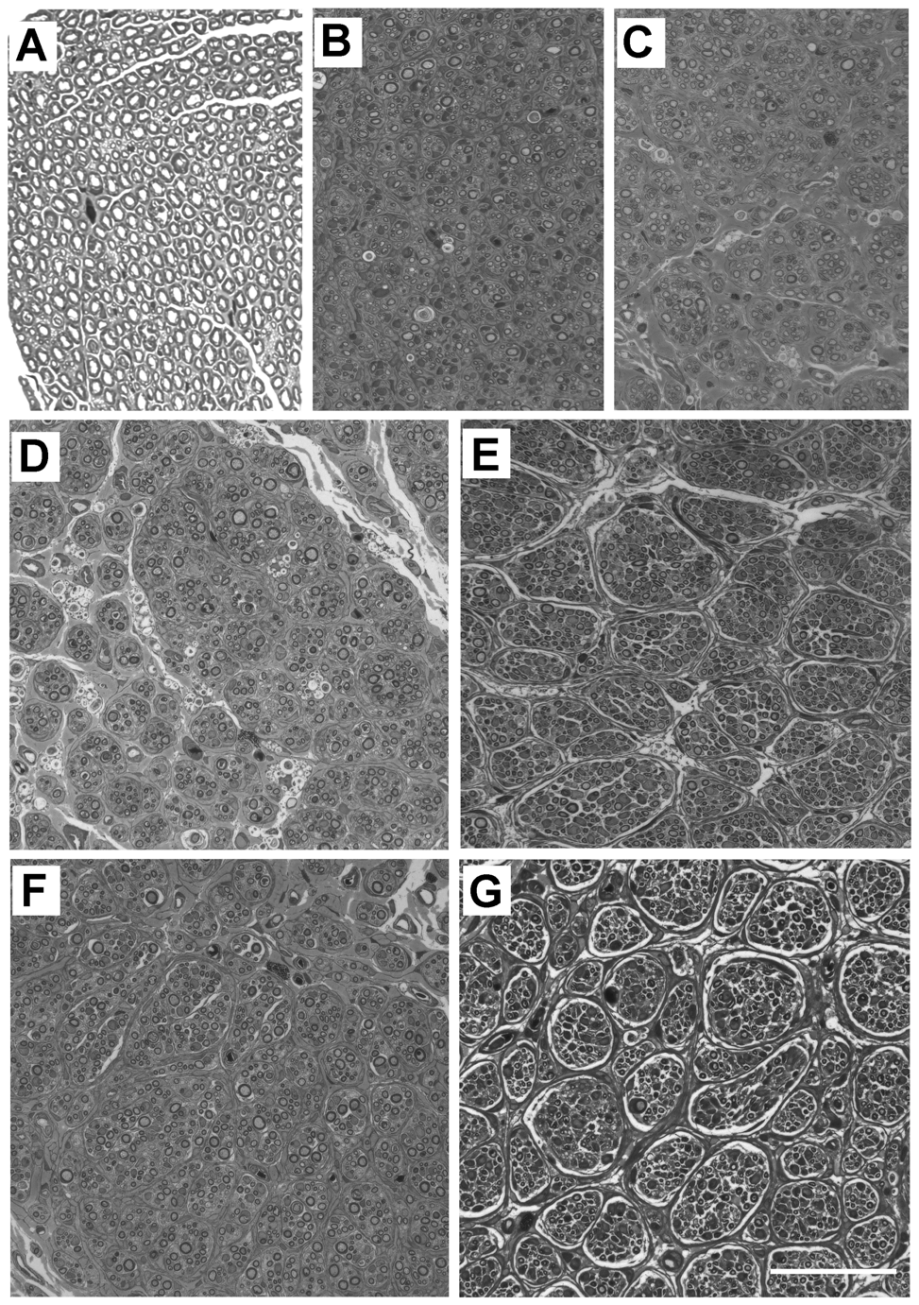
Fascicular architecture in grafts. Semi-thin sections of a normal nerve (A), an autograft (B), and acellular (C), SCs (D), BDNF (E), CNTF (F) and NT3 (G) grafts. Note the pronounced fascicular organization in SC reconstituted grafts, especially in E and G. Scale bar for A–G: 50 µm.

Semi-thin cross-sections from the distal end of each graft and normal PN were collected for assessment of overall tissue architecture and fascicular morphology. Normal nerves were mostly homogenous, with many evenly distributed, large myelinated axons and no clear fascicular demarcation (Fig. 7A). On the other hand, grafted nerves (Fig. 7B–G) consistently contained discernible fascicles and small axonal bundles. Both autografts (Fig. 7B) and acellular grafts (Fig. 7C) had the most homogenous appearance, with compact fascicles. However in grafts containing unmodified SCs the fascicular organization was slightly more obvious (Fig. 7D) and in all SC-neurotrophic factor grafts large fascicles were clearly demarcated. This was obvious in grafts that had been reconstituted with BDNF (Fig. 7E) and particularly evident in grafts containing NT3 expressing SCs (Fig. 7G). The minimum diameter fascicles in unmodified SCs and CNTF grafts was not significantly different from each other (11.8 and 11.2 µm respectively); however fascicles in BDNF and NT3 grafts were significantly larger (Bonferroni, p<0.05, mean diameters of 17.8 and 19.0 µm respectively).

### Morphology of Remak bundles

**Figure 8:**
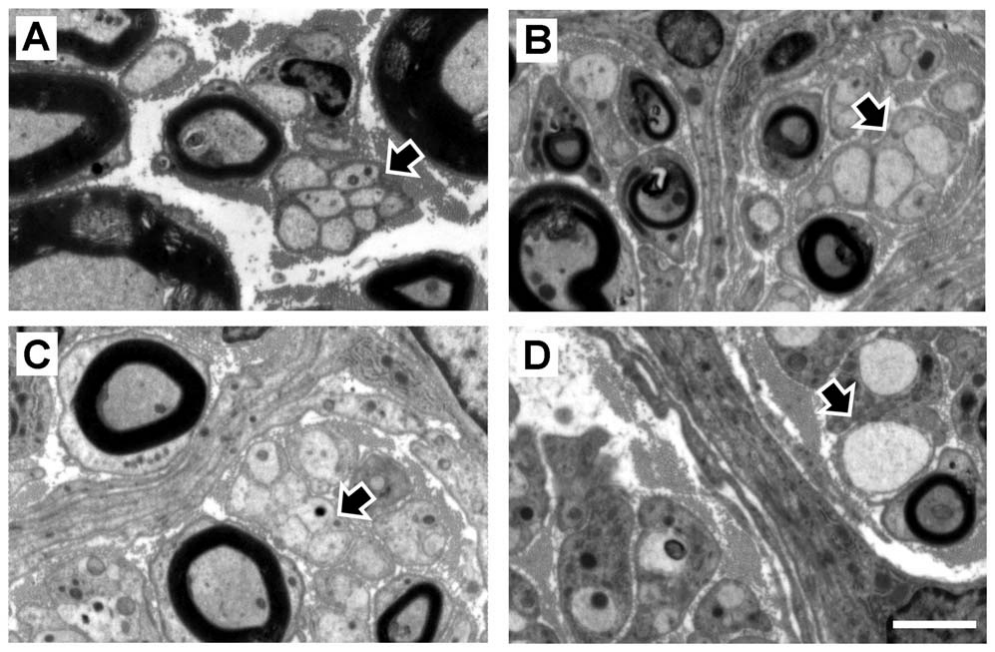
Unmyelinated axons in Remak bundles. These axons (black arrows) are shown in representative electron micrographs of normal nerve (A), BDNF (B), CNTF (C) and NT3 (D) grafts. Scale bar for A–D: 2.5 µm.

The organization of unmyelinated profiles into Remak bundles [42]–[45] in grafted and normal PN was examined in electron micrographs (Fig. 8). Normal peroneal nerves contained many large myelinated axons as well as clearly demarcated Remak bundles containing any number of very tightly grouped unmyelinated axons surrounded by a single SC (arrow in Fig. 8A, see also Table 1). In normal PN, unmyelinated axons in each bundle were relatively homogenous in size whereas in grafts the variation was much greater and Remak bundles were less compact (arrow in Fig. 8B and 8C). The large number of relatively large unmyelinated axons was noteworthy in BDNF and particularly in NT3 grafts, where some axons were larger than adjacent myelinated axons (arrows in Fig. 8B and 8D).

**Table 1:** Number of axons in each Remak bundle.

	Normal	Autograft	Acellular	SC	BDNF	CNTF	NT3
**Mean**	3.53	3.47	4.52	2.31	1.86	2.81	2.36
**SEM**	0.397	0.249	0.389	0.192	0.130	0.203	0.161
**Variance**	12.8	5.5	14.7	3.9	1.5	4.2	4.0
**Minimum**	1	1	1	1	1	1	1
**Maximum**	17	11	18	10	7	12	13
**1^st^ quartile**	1	2	2	1	1	1	1
**Median**	2	3	3	1	1	2	2
**3^rd^ quartile**	4.5	4	6	3	2	3	3

Descriptive statistics of the number of unmyelinated axons in Remak bundles, including mean and standard error of mean (SEM), variance and range of data distribution represented by minimum and maximum number of axons, and percentiles distribution, namely first, second (median) and third quartile. There were significant differences between experimental groups in the median number of unmyelinated axons in Remak bundles (p<0.0005), with significantly higher numbers in autografts and acellular grafts than in NT3, SCs and BDNF grafts. Moreover, the number of unmyelinated axons in the latter was also significantly lower than in normal nerves and in CNTF grafts. Further details on statistical analysis provided as Statistical Information S1.

### Density of unmyelinated axons in Remak bundles

**Figure 9:**
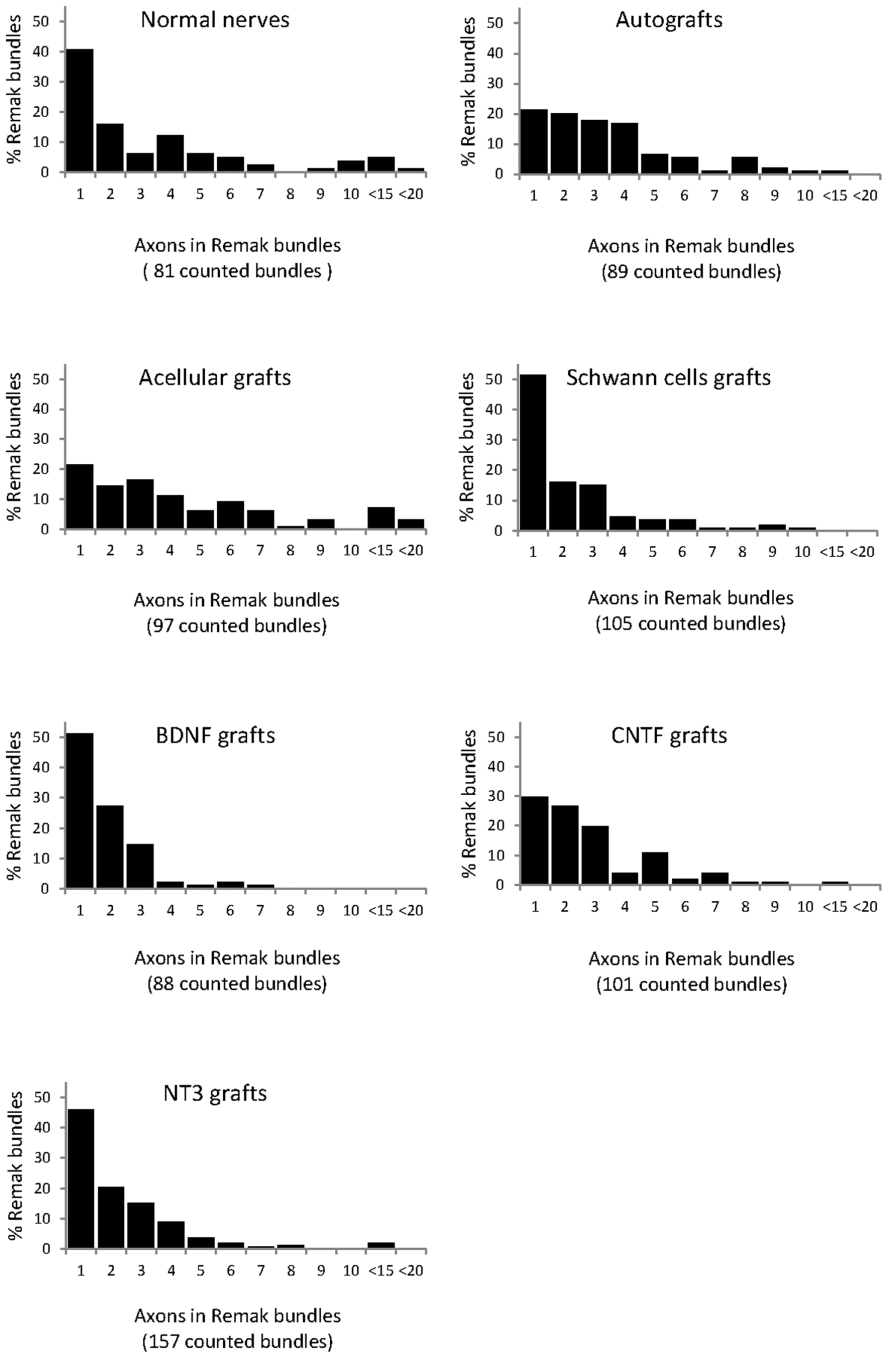
Occurrence and number of axons in Remak bundles, with pooling of counts between 10–15 and 15–20 axons in bundles.

The number of axons in each Remak bundle was quantified (Table 1). The highest mean was found in the acellular group and the lowest in the BDNF group. In the latter group there was also the lowest maximum number of unmyelinated axons in a Remak bundle, while the highest maximum was in the acellular group, similar to the number in normal nerves. There were significant differences between experimental groups (Kruskall-Wallis, p<0.0005), and a Dunn's post-hoc test revealed that the median number of unmyelinated axons in Remak bundles was significantly greater in acellular and autografts than in SCs, NT3 and BDNF grafts. The numbers in BDNF were also significantly lower than in normal PN and CNTF grafts. The frequency of axon numbers in each bundle was expressed as a percentage of the total number of counted bundles (Fig. 9). Strikingly, there were almost twice as many Remak bundles in NT3 grafts (total of 157) than in normal PN (total of 81) in similar quantified areas. Bundles containing only a single axon were most common in normal PN and in SC, BDNF and NT3 grafts.

### Myelinated axons

**Figure 10:**
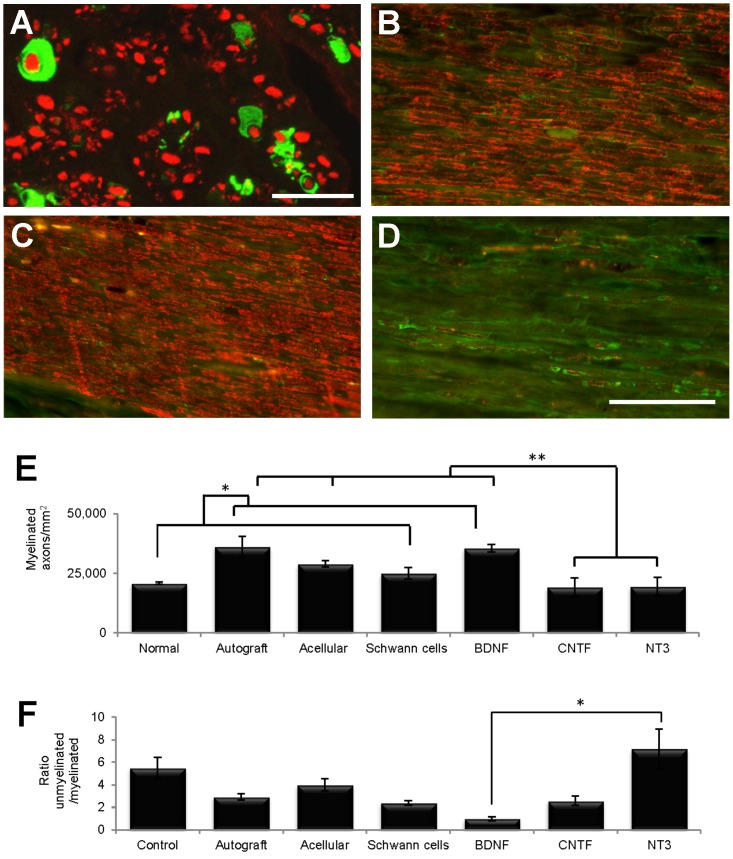
Myelination of regenerating axons within grafts. (A) In the pilot sciatic nerve experiment, cross-sections from the middle of GFP-SC grafts showed profiles with typical SC morphology (green) surrounding axons labelled with PanNF (red). (B–D) In the main peroneal graft experiment, comparison of longitudinal sections immunostained with S100 (green) and MBP (red) showed that normal nerves (B) had less myelin than autografts (C) and more myelin than NT3 grafts (D). (E) Quantification of semi-thin sections revealed that the number of myelinated axons/mm^2^ in normal nerves and SCs grafts was significantly lower (*) than in autografts and BDNF grafts, and the numbers in the latter two and in acellular grafts were significantly greater (**) than in CNTF and NT3 grafts. (F) The ratio of unmyelinated to myelinated axons in the BDNF group was significantly less than in the NT3 group. Values represent M ± SEM of n = 3; p<0.05. Further details on statistical analysis provided as Statistical Information S1. Scale bars: 10 µm in A; 100 µm for B–D.

In our pilot sciatic nerve graft study, LV-GFP transduced SCs with typical adult morphologies were seen wrapping either one or several regenerate PanNF^+^ axons at 8 weeks after transplantation (Fig. 10A). In the main peroneal nerve graft experiment, SC content was also confirmed in longitudinal sections of normal PN and in grafts by immunostaining with the marker S100 (Fig. 10B–D). Myelin content was assessed by co-immunostaining sections with an MBP antibody. There was clear myelin staining associated with SC profiles in normal nerves (Fig. 10B) and an even more conspicuous amount of myelin in sections of autografts (Fig. 10C). In contrast there was very little stained myelin in NT3 graft sections (Fig. 10D).

Myelinated axons were counted in semi-thin cross-sections (see Fig. 7), their density calculated and assessed with a one-way analysis of variance. Post-hoc comparisons using LSD confirmed the qualitative immunohistochemical survey in that autografts and BDNF grafts contained significantly more myelinated axons/mm^2^ than normal nerves and SC grafts. In addition, autografts, acellular and BDNF grafts contained more myelinated axons/mm^2^ than CNTF and NT3 grafts (Fig. 10E). The density of unmyelinated axons did not differ between groups (data not shown), however the ratio of unmyelinated to myelinated axons did differ significantly (Kruskall Wallis, p = 0.024)(Fig. 10F). In particular, there was a lower ratio of unmyelinated to myelinated axons in grafts containing BDNF compared to NT3 expressing SCs, indicating that these two neurotrophins had opposing influences on the myelination of regenerated peripheral axons. Note that the unmyelinated to myelinated axon ratio in our normal group is similar to the ratio previously reported for normal cutaneous nerves [43].

### G-ratios

**Figure 11:**
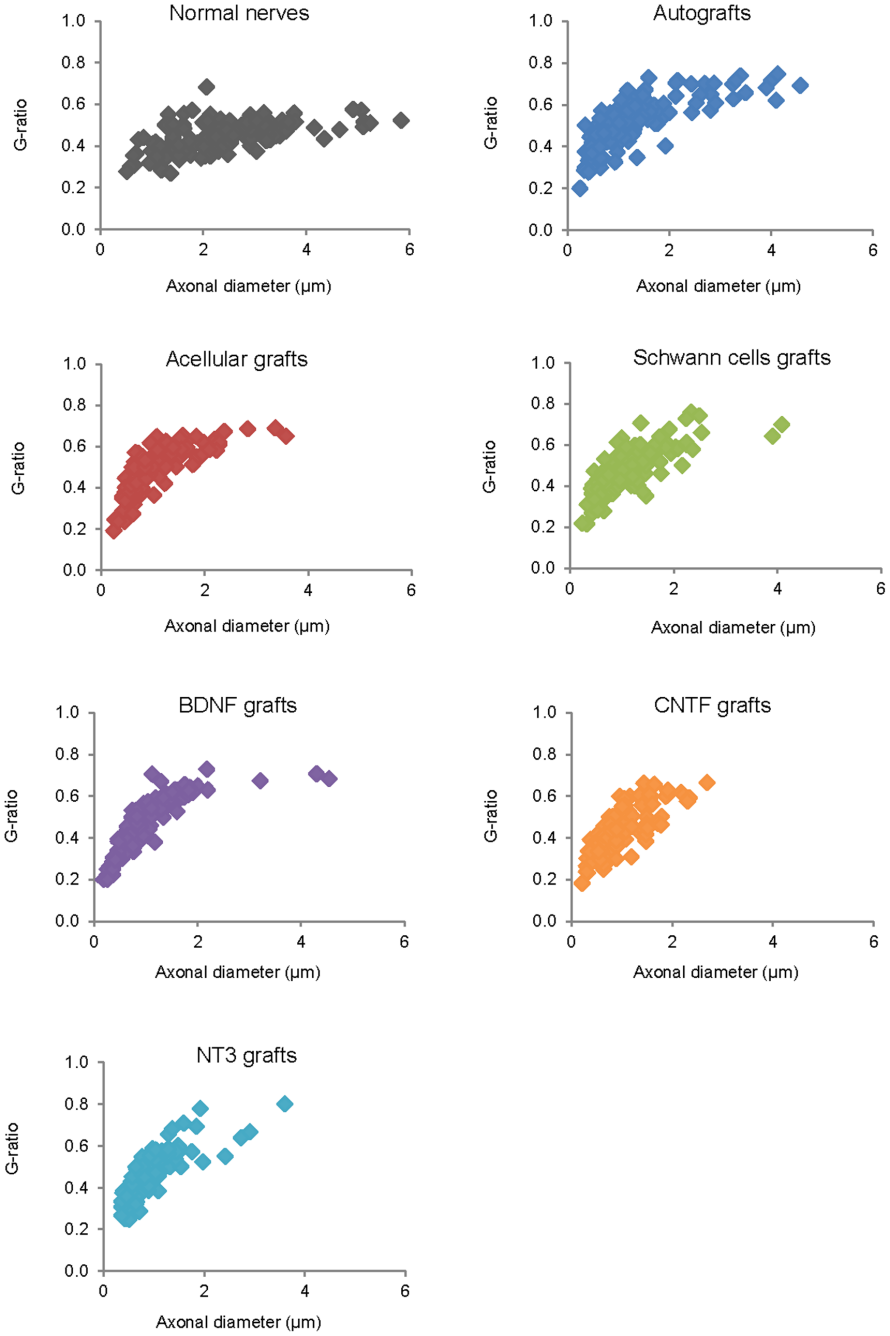
G-ratios of myelinated fibers in each experimental group (120 fibers measured in each group). Note the greater number of large diameter axons in normal nerve and in autografts, the G-ratio in the latter being the highest of all experimental groups and significantly higher than in the normal, BDNF, CNTF and NT3 groups. The G-ratio values were also significantly lower in normal nerves compared to acellular grafts and significantly higher in SC compared to CNTF grafts (see also Table 3). Further details on statistical analysis provided as Statistical Information S1.

The area of individual myelinated axons was larger in normal PN compared to all graft groups (Table 2). Furthermore, the average area of myelinated axons in autografts was significantly greater than in BDNF and NT3 grafts. From the diameter of myelinated axons and myelinated fibers, the latter including the myelin sheath, the G-ratio was calculated (dividing the former by the latter) (Fig. 11). The lower the G-ratio, the greater the thickness of the myelin sheath around the axon, and generally speaking, the smaller the axon caliber the lower the G-ratio [46]. Descriptive statistics showed that the highest mean and median G-ratio was in autografts, followed by acellular, SCs, BDNF and NT3 grafts, all of which had higher means and medians than normal PN. CNTF grafts contained axons with the lowest average G-ratio amongst all experimental groups (Table 3). G-ratios differed between groups (Kruskall-Wallis, p<0.0005), and a Dunn's post-hoc test revealed that the G-ratio was significantly lower in normal PN compared to both acellular and autografts. In the latter, the G-ratio was higher than in any neurotrophic factor group, and values in the CNTF group were lower than in the SC group.

**Table 2:** Area of individual myelinated axons.

	Normal	Autograft	Acellular	SC	BDNF	CNTF	NT3
**Mean**	9.5	3.6	2.1	2.0	1.9	1.9	1.6
**SEM**	0.633	0.406	0.209	0.175	0.233	0.202	0.163
**Variance**	48.1	19.8	5.2	3.7	6.5	4.9	3.2
**Minimum**	0.65	0.14	0.19	0.16	0.16	0.14	0.20
**Maximum**	33.00	25.90	12.90	12.20	18.80	13.70	10.96
**1^st^ quartile**	4.15	0.78	0.68	0.76	0.63	0.67	0.49
**Median**	8.12	1.94	1.30	1.36	1.18	1.31	1.03
**3^rd^ quartile**	13.15	4.66	2.67	2.51	2.18	2.46	2.00

Descriptive statistics of areas of myelinated axons, including mean and standard error of mean (SEM), variance and range of the distribution represented by minimum and maximum, and percentile distribution, namely first, second (median) and third quartile.

**Table 3:** G ratios.

	Normal	Autograft	Acellular	SC	BDNF	CNTF	NT3
**Mean**	0.44	0.52	0.49	0.48	0.46	0.43	0.46
**SEM**	0.007	0.011	0.01	0.01	0.012	0.01	0.01
**Variance**	0.006	0.014	0.013	0.012	0.017	0.011	0.013
**Minimum**	0.268	0.20	0.191	0.214	0.20	0.18	0.25
**Maximum**	0.681	0.75	0.689	0.757	0.73	0.66	0.80
**1^st^ quartile**	0.40	0.44	0.40	0.40	0.37	0.36	0.38
**Median**	0.45	0.52	0.50	0.46	0.45	0.42	0.45
**3^rd^ quartile**	0.50	0.60	0.58	0.56	0.57	0.50	0.55

Descriptive statistics of G-ratios in each group including mean and standard error of mean (SEM), variance and range of the distribution with minimum and maximum values, as well as percentile distribution, including first, second (median) and third quartile. Further details on statistical analysis provided as Statistical Information S1.

### Behavioral gait analysis

**Figure 12:**
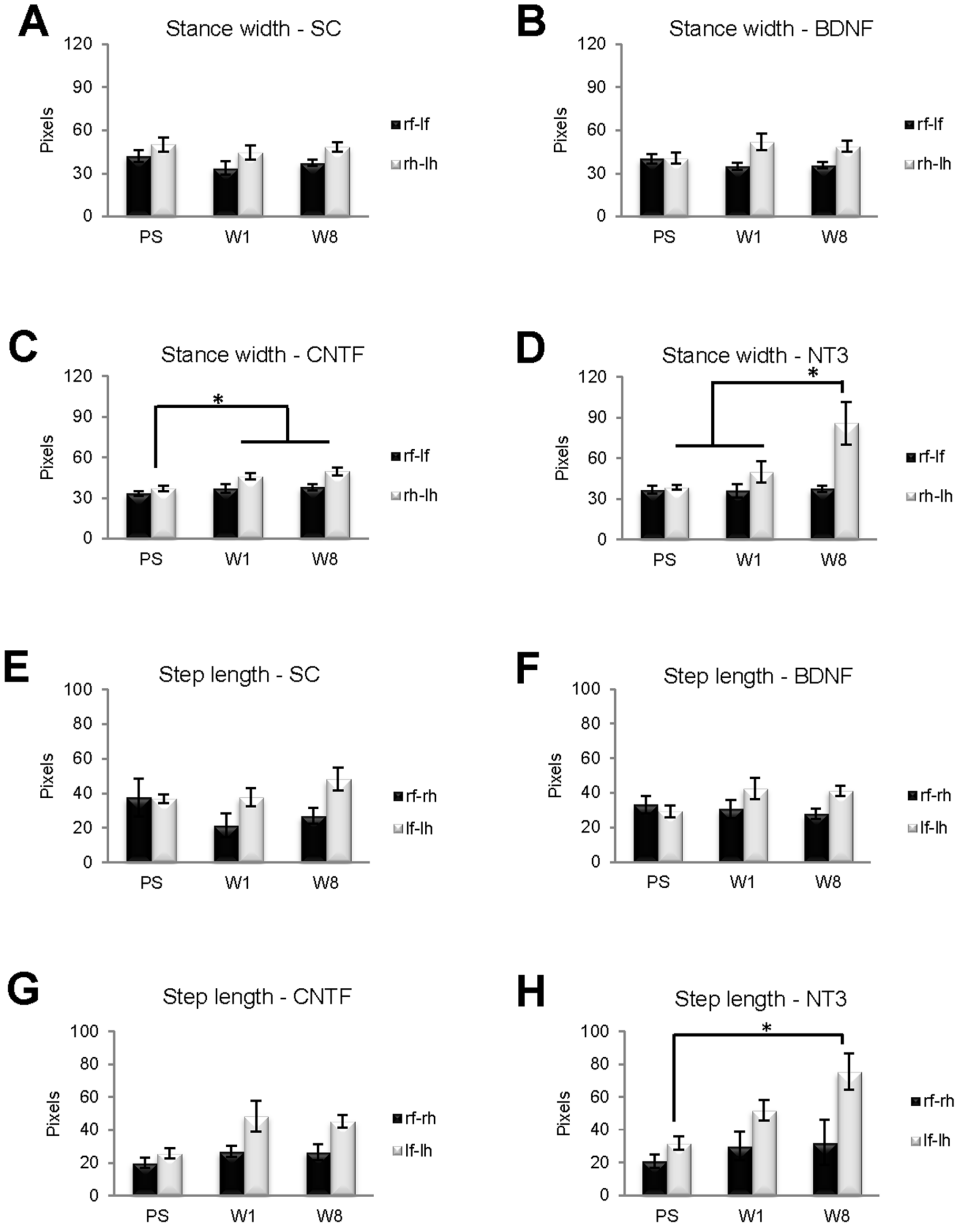
Locomotor function of rats from SC, BDNF, CNTF and NT3 groups prior to surgery (PS), and at one (W1) and eight (W8) weeks after surgery, was analyzed from digitized recordings using the Ratwalk® software. Two quantitative parameters generated by this software were analyzed: stance width (A–D) and step length (E–H). There were significant differences in both parameters in distances involving the injured left hindlimbs. Namely, the stance width of hindlimbs (rh-lh) in the CNTF group significantly increased from PS (* in C) to W1 and W8, and in the NT3 group it was significantly greater in W8 (* in D) than in both W1 and PS. Regarding the step length on the left (lf-lh), in the NT3 group there was a significant increase (* in H) from PS to W8. There were no significant differences in either the SC or BDNF groups on the two gait parameters analyzed. Values represent M ± SEM of n = 4; p<0.05.

To assess the impact of neurotrophic factors on functional recovery, the Ratwalk® system was used to analyze gait parameters in four PN graft groups: SCs, BDNF, CNTF, NT3. This software was developed for assessing recovery of function after spinal cord injuries, thus a pilot study was conducted using animals with sciatic nerve injury to determine which walking parameters might be the most informative in evaluating recovery after PN injury. Consistent with a previous study [47], a significant difference in the mean distance of step length was detected on the injured side (data not shown), and accordingly it was one of the parameters selected for analysis in this experiment. Others [48], reported that the distance between hindlimbs is reduced after sciatic nerve injury, and there is also abnormal foot rotation [49], thus stance width was also selected for analysis. In each animal, stance width and step length were analyzed prior to surgery (PS), and one (W1) and eight (W8) weeks after PN transplantation (Fig. 12). Rats were randomly ascribed to different experimental graft groups before surgery. These normal, PS animals displayed variation in stance width and step length, such that a comparison of these parameters between groups failed to reveal significant differences for either forelimb stance width, or step length on the left side.

Stance width, the mean distance between the two forelimbs (rf-lf) or between the two hindlimb (rh-lh), increased significantly from PS to W1 (p = 0.038) and W8 (p = 0.007) in hindlimbs (rh-lh) of animals from the CNTF group. In the NT3 group, stance width of hindlimbs (rh-lh) was significantly greater in W8 than in both W1 (p = 0.034) and PS (p = 0.009).

Step length is the distance between ipsilateral limbs, comprising the mean distance between right forelimb (rf) and right hindlimb (rh), or the mean distance between left forelimb (lf) and left hindlimb (lh). In the NT3 group the step length on the left, grafted side (lf-lh) increased significantly from PS to W8 (p = 0.01). There was a trend associated with the step length on the left side (lf-lh) in rats from the CNTF group, which almost reached significance (p = 0.058).

## Discussion

Using a new approach combining *ex vivo* gene therapy of SCs with transplantation we examined whether neurotrophic factors delivered via LV-modified SCs in reconstituted PN grafts is a useful strategy to enhance PN regeneration. In the present series of experiments we examined the impact of neurotrophic delivery on graft morphology, axonal regeneration, myelination and functional recovery after unilateral peroneal nerve injury. The three factors chosen (BDNF, CNTF and NT3) have all previously been shown to be important in various aspects of PN regeneration [50]–[57]. Using LV-GFP constructs it was found that over 90% of SCs continued to express GFP *in vitro* 48 h after transfection [28], and here we confirmed *in vivo* that GFP-expressing SCs colonized the entire length of grafts and remained viable for at least 8 weeks post-transplantation. It is important to note that, using some of these same vectors, previous *in vitro* and *in vivo* work has shown sustained expression of mRNA for growth factor transgenes, and neurotrophic factors continue to be expressed and secreted by LV-transduced SCs *in vitro* and in PN *in vivo* [22], [23], [28], [29].

Most graft types supported axonal regrowth at similar densities, although CNTF grafts possessed the fewest axons. NT3 grafts were notable in containing a high density of IB4 and CGRP labelled sensory axons when compared to other graft types, including autografts. In terms of morphology, fascicles of axons were especially evident in grafts containing SCs expressing BDNF or NT3. The proportion of myelinated axons was highest in BDNF grafts and lowest in NT3 grafts. Consistent with the morphological data, functional analysis of stance width and step length revealed changes in the locomotor performance of rats in the CNTF and especially NT3 groups. Such changes were associated with the operated left hindlimb and were not observed in the control SC group. An overview of the effects of the tested neurotrophic factors on each of the assessed regenerative parameters is presented in the summary Table (Table 4) and will be further discussed below.

**Table 4:** Summary of data.

		BDNF	CNTF	NT3
**Graft morphology**	*Section width*	Significantly wider than normal nerves	Wider than normal nerves	Widest of all and significantly wider than others
	*Fascicles*	Clearly apparent	Apparent	Clearly apparent
	*Remak bundles*	Fewer than in other NTF grafts		Highest number
**Axonal regeneration**	*βIII-Tubulin*		Fewer than in all other grafts	Lowest number of axons/mm^2^
	*PanNF*			Low number
	*IB_4_*	Low number		Highest number
	*CGRP*	Low number		Highest number
**Myelination**	*Unmyelinated axons*	Lowest number and lower than normal nerves		
	*Myelinated axons*	More than in normal nerves or other NTF grafts	Low density but with high mean area of myelin	Fewer than in normal nerves and any other grafts
	*Ratio of unmyelinated to myelinated*	Lowest, and significantly lower than NT3		Highest, and significantly higher than BDNF
	*G-ratio*	High	Lowest of all groups	High
**Functional recovery**	*Stance width of hindlimbs*		Significantly increased from PS to W1 and W8	Significantly greater at W8 than PS and W1
	*Step length on the left*			Significant increase from PS to W8

General overview of tested neurotrophic factors (NTF), which were BDNF, CNTF and NT3, on the various regenerative parameters examined, namely, grafts morphology, axonal regeneration, myelination and functional recovery. The latter included three time-points: pre-surgery (PS), one week (W1) and eight weeks (W8) after surgery.

### Regeneration in PN grafts containing LV-modified Schwann cells

Host nerve stumps distal to NT3 grafts were the widest and the density of βIII-Tubulin^+^ axons/mm^2^ was lowest in this group. Factors that may have influenced the cross-sectional area of distal host nerve stumps include axonal number, extent of myelination, immune and glial cell infiltration and/or proliferation, and amount of extracellular matrix. Within most grafts there were significantly less axons counted proximally versus distally, perhaps due to local sprouting and/or axons becoming entangled within grafts as they approached the distal suture area. NT3 grafts themselves were wider than normal peroneal nerve and other graft types, in accordance with reported effects of this neurotrophin on increased proliferation [58], migration [59] and survival [60] of SCs in grafts. Injury-induced NT3 levels increase in the sciatic nerve up to a month after ventral root avulsion and reimplantation [61], thus expression of NT3 by SCs within reconstituted grafts may have enhanced local axonal sprouting, known to occur during regeneration [3], [57]. NT3 grafts also possessed high laminin immunoreactivity and at EM level showed signs of increased extracellular matrix deposition, especially evident between, and surrounding, the large fascicular bundles (eg Fig. 7G). In counts from longitudinal sections, CNTF grafts contained the least number of axons, a surprising outcome given that this neurotrophic factor is thought to play an important role after PN injury [62], [63]. Interestingly, these grafts contained a large number of ED1 positive macrophages/monocytes, consistent with previous reports that CNTF is a chemotactic agent for these cells [64], [65].

In some graft groups there were intriguing differences in axonal counts in near adjacent longitudinal sections immunostained for either βIII-Tubulin or PanNF. Counts of PanNF and βIII-Tubulin stained profiles/mm were similar in normal nerves, however there were consistently fewer PanNF^+^ fibers within grafts. The neurofilament proteins identified by PanNF are related to axonal caliber, with increases in axonal diameter generally correlating with increased neurofilament content [66]. This makes PanNF a marker particularly suitable for identifying axons of large caliber. After axotomy the slow transport of neurofilaments along axons is associated with a smaller caliber of regenerated axons, which could explain the reduction in the number of PanNF^+^ profiles in all grafts. βIII-Tubulin expression selectively increases during axonal regrowth and is added near the tip of regenerating axons [67]–[70], thus making βIII-Tubulin a more general marker for regenerating axons, irrespective of their caliber. It is therefore of interest that axonal counts using βIII-Tubulin immmunohistochemistry were significantly greater in acellular, untransduced SC and NT3 grafts, perhaps indicative of a greater proportion of small caliber regenerating sprouts in these particular grafts.

### IB_4_ and CGRP counts

The number of axons positive for IB_4_ was greatest in NT3 grafts. IB_4_^+^ sensory neurons are small nonpeptidergic nociceptors with unmyelinated axons, responsive to glial cell-derived neurotrophic factor (GDNF) but not nerve growth factor (NGF) [71], and express tyrosine kinase RET and subunits of the GDNF receptor family [72]. They have been reported to be vulnerable to injury and have poor regenerative capacity [34], [73]. In adults, NT3 and GDNF rescue specific subpopulations of DRG neurons [74], [75], but to our knowledge IB_4_^+^ neurons do not express trkC receptors [76]. *In vitro*, NT3 has little effect on neurite expression from cultured IB_4_^+^ adult sensory neurons and may even have growth-inhibitory effects [76]. Further work is needed to determine if the observed effects of NT3 in our *in vivo* model are indirect, perhaps acting via host cells that colonize the grafts.

CGRP is found in peptidergic, unmyelinated, nociceptive, sensory neurons, expressing substance P [23], [41], and has also been reported to be present in motor neurons [77]. DRG neurons expressing CGRP are responsive to CNTF, GDNF and NGF, and express trkA and p75 receptors [72], and the peptide is up-regulated after PN injury [78]. The highest numbers of CGRP^+^ axons was found in NT3 grafts and the lowest in BDNF grafts. Again, the mechanisms underlying these effects are unclear, although *in vitro*, BDNF has been reported to inhibit neurite outgrowth from cultured adult sensory neuron populations [76].

### Fascicular architecture and Remak bundles

Interestingly, unlike normal PN, a clear fascicular organization was evident in grafts, particularly those containing SCs expressing BDNF or NT3. Demarcation of fascicles appeared to result from intra- and inter-fascicular deposition of collagen and from loosely organized Remak bundles. All graft types were more heterogeneous in structure than normal PN, perhaps due to greater amounts of extracellular matrix, but also the relative absence of myelin may allow for greater plasticity within regenerated nerves [44].

In normal PN, unmyelinated axons were closely wrapped by a Remak SC, but in grafts the bundles were less compact and contained variable numbers of unmyelinated profiles, the highest average number in acellular grafts and the lowest in BDNF grafts. However in the latter and in NT3 grafts there were numerous relatively large unmyelinated axons, suggesting that besides axon caliber [79] these factors play an important role in regulating myelination during regeneration. Axonal areas were comparatively small in BDNF grafts, nonetheless most axons were myelinated. Indeed, BDNF grafts contained a greater density of myelinated axons compared to normal PN and they contained the lowest median ratio between unmyelinated and myelinated axons, consistent with an enhanced myelination profile after BDNF delivery [53], [55], [80]. Mice lacking BDNF possess fewer myelinated axons [81], and BDNF antibodies reduce the number and density of myelinated fibers after sciatic nerve injury [52].

### Myelination

NT3 grafts contained an average unmyelinated to myelinated axon ratio of 8∶1. Unmyelinated axon numbers per bundle were relatively low, however the overall density of Remak bundles was greatest in these grafts. The presence of large diameter but unmyelinated axons in each Remak bundle, together with the smaller proportion of myelinated axons, supports the view that NT3 inhibits SC myelination [82], [83]. Grafts containing CNTF expressing SCs also had low myelinated axon densities, however the mean area of myelin per axon was greater in the CNTF group compared to axons in BDNF grafts, indicating different effects on myelination from each of these factors. G-ratios in BDNF grafts were greater than in normal PN, suggesting that BDNF enhanced myelination by increasing the number of axons that were myelinated, not necessarily by increasing the amount of myelin around a given axon. On the other hand, myelinated axons in CNTF grafts had a relatively small average G-ratio. Given that axonal areas were significant smaller in CNTF grafts compared to normal PN, these data indicate that the relative thickness of myelin around each axon had increased, consistent with a role for CNTF in PNS myelination [55], [84].

### Functional analysis

We tested overall locomotor function after grafts to the mixed peroneal nerve because, after PN injuries in rodents, assessment of functional recovery is difficult, particularly when measuring discrete and specific somatosensory properties, due to the need for repetitive and accurate stimulation of the same skin region, overlapping innervation fields [85] and because the fields change after nerve injury [86]. Walking patterns were analyzed using the Ratwalk® system, which is similar to the Catwalk system and allows the objective and quantitative assessment of dynamic and static gait parameters [30], [87]–[89]. Stance width was wider between hindlimbs than between forelimbs, even before the injury. However in the group with CNTF grafts hindlimb stance width was increased at 1 and 8 weeks after injury, and in the NT3 group the distance between hindlimbs was significantly increased at 8 weeks. At the latter time the step length between left forelimb and injured left hindlimb was also greater in the NT3 group. Others have reported increased step length on the injured side after peroneal nerve crush [47], although in the earlier study step length distance returned to normal after 22 days, whereas here an altered gait was still a feature of NT3 grafted rats 8 weeks post-injury, probably due to differences in the type of injury.

NGF and associated receptors are generally thought to be important in neuropathic pain responses after PN injury [90], [91], although recent work has suggested that NT3 may also play a role in hypersensitivity and pain, associated with the sprouting of sensory nerves after skin injury [92]. There was a significant increase in the number of (nociceptive) IB4^+^ axons in our NT3 grafts and we noted in these rats that the paw-prints from the injured left hindlimb were often light and barely distinguishable, suggestive of decreased pressure in the left hindlimb. After repair of peroneal defects, autotomy was only infrequently observed and did not vary between graft groups, suggesting no severe changes in sensory responsiveness; nonetheless any altered mechanosensitivity in the injured left hindlimb could have contributed to the changes in stance and step length seen during locomotion.

In conclusion, our novel method of using LV-engineered SCs in chimeric bridging grafts to deliver targeted neurotrophic support to regenerating axons after PN injury has revealed that each factor has a spectrum of effects on the overall regenerative process. There were differences in graft morphology, extent of myelination and type of axon regenerating through the grafts. Functional differences were also apparent. Given that SC phenotype differs in motor versus sensory nerves [4], and different axonal populations have different neurotrophic requirements, this new approach using genetically modified SCs in reconstituted bridges may permit more selective and effective stimulation of sub-populations of motor or sensory neurons after defined injury to a particular PN or branch of the nerve.

## Supporting Information

Statistical Information S1 Supporting Statistical Information
(DOCX)
