# Supplementary material for: Immunohistochemical, Ultrastructural and Functional Analysis of Axonal Regeneration through Peripheral Nerve Grafts Containing Schwann Cells Expressing BDNF, CNTF or NT3
Source: PLoS One. 2013 Aug 9;8(8):e69987. doi: 10.1371/journal.pone.0069987 (PMC3739754; doi:10.1371/journal.pone.0069987)
Supplement: Statistical Information S1 — Supporting Statistical Information (DOCX) [file pone.0069987.s001.docx]

**Supporting Statistical Information**

S-Figure3

(E) Cross-sectional area of nerves and grafts and (G) the ratio of βIII-Tubulin^+^ axons/mm^2^ between Block A and E were compared using a non-parametric Kruskall-Wallis test, revealing significant differences between groups (in both cases p<0.0005). Median values of each data set were calculated and a Dunn’s post-hoc test with all pairwise comparisons identified which groups differed. (F) The number of βIII-Tubulin^+^ axons/mm^2^ in Block A and E were calculated and square root transformed to correct heterogeneity of variance, although this did not correct the non-normality, according to a Saphiro-Wilk test at p<0.05 (W=0.987, p=0.04). However, given the high p-value, and with quartile-by-quartile (Q-Q) and boxplots revealing only one outlier value, data were considered “normal”. Data was analysed using a linear mixed model, which considered experimental group and tissue block as fixed effects and animal within group a random effect. The use of this statistical model allowed to simultaneously compare Block A with Block E and to compare experimental groups. Accordingly the model included a Sidak adjustment for multiple comparisons.

S-Figure4

(A) Given the non-normality of the width of sections from Block C data two non-parametric tests were used, namely, a Friedman test to compare counting distances (prox/med/dist) within each group, and a Kruskall-Wallis test to compare experimental groups. To compensate for multiple testing of the same data a Bonferroni adjustment was applied to the significance level (which became p<0.025). (B) The number of βIII-Tubulin^+^ axons/mm was analysed using a linear mixed model, considering experimental group and tissue block as fixed effects and animal within group a random effect. This model simultaneously compared axonal numbers at each counting distance within each group and compared experimental groups, including a Sidak adjustment in the model for multiple comparisons.

S-Figure5

(G) A linear mixed model was used to simultaneously compare the number of axons/mm identified with βIII-Tubulin and with PanNF, and to compare experimental groups. The statistical model considered each axonal marker and each experimental group, as well as the interaction between these two parameters, as fixed effects, and considered animal within group as a random effect. A Sidak adjustment was included in the model to adjust for multiple comparisons of the same data.

S-Figure6

(D) Data for the number of IB4^+^ axons/section and (E) for the number of CGRP^+^ axons/section did not follow a normal distribution. Therefore each data set was logarithmically transformed before being analysed with a one-way analysis of variance to look for significant differences between groups, and with LSD post-doc tests to specifically identify groups which significantly differed.

S-Figure10

(E) The density of myelinated axons/mm^2^ was tested using a one-way analysis of variance in order to assess statistically significant differences between experimental groups, and LSD post-hoc comparison to specifically identify which groups differed. (F) The ratio between unmyelinated and myelinated axons was calculated dividing the former by the latter. Ratios were compared using a Kruskal-Wallis to test for significant differences between experimental groups, a subsequently a Dunn’s post-hoc test with all pairwise comparisons specifically identified which groups were significantly different.

S-Figure11 and S-Table3

G-ratios were compared using a Kruskal-Wallis test to look for significant differences between experimental groups, which were subsequently identified using a Dunn’s post-hoc test with all pairwise comparisons.

S-Table1

The numbers of axons in Remak bundles were compared using a Kruskall-Wallis test to look for significant differences between experimental groups, followed by a Dunn’s post-hoc test with all pairwise comparisons to specifically identify which groups differed.
